# Increased prevalence of the *pfdhfr/phdhps *quintuple mutant and rapid emergence of *pfdhps *resistance mutations at codons 581 and 613 in Kisumu, Kenya

**DOI:** 10.1186/1475-2875-9-338

**Published:** 2010-11-24

**Authors:** Maroya D Spalding, Fredrick L Eyase, Hoseah M Akala, Sheryl A Bedno, Sean T Prigge, Rodney L Coldren, William J Moss, Norman C Waters

**Affiliations:** 1 Department of Epidemiology, Johns Hopkins Bloomberg School of Public Health, Baltimore, Maryland, USA; 2 Global Emerging Infections System (GEIS), United States Army Research Unit-Kenya Walter Reed/KEMRI project, Kisumu, Kenya; 3 Department of Preventive Medicine and Biometrics, Uniformed Services University of the Health Sciences, Bethesda, Maryland, USA; 4 Department of Molecular Microbiology and Immunology, Johns Hopkins Bloomberg School of Public Health, Baltimore, Maryland, USA; 5 Walter Reed Army Institute of Research, Silver Spring, Maryland, USA

## Abstract

**Background:**

Anti-malarial drug resistance in Kenya prompted two drug policy changes within a decade: sulphadoxine-pyrimethamine (SP) replaced chloroquine (CQ) as the first-line anti-malarial in 1998 and artemether-lumefantrine (AL) replaced SP in 2004. Two cross-sectional studies were conducted to monitor changes in the prevalence of molecular markers of drug resistance over the period in which SP was used as the first-line anti-malarial. The baseline study was carried out from 1999-2000, shortly after implementation of SP, and the follow-up study occurred from 2003-2005, during the transition to AL.

**Materials and methods:**

Blood was collected from malaria smear-positive, symptomatic patients presenting to outpatient centers in Kisumu, Kenya, during the baseline and follow-up studies. Isolates were genotyped at codons associated with SP and CQ resistance. *In vitro *IC_50 _values for antifolates and quinolones were determined for isolates from the follow-up study.

**Results:**

The prevalence of isolates containing the *pfdhfr *N51I/C59R/S108N/*pfdhps *A437G/K540E quintuple mutant associated with SP-resistance rose from 21% in the baseline study to 53% in the follow-up study (p < 0.001). Isolates containing the *pfdhfr *I164L mutation were absent from both studies. The *pfdhps *mutations A581G and A613S/T were absent from the baseline study but were present in 85% and 61%, respectively, of isolates from the follow-up study. At follow-up, parasites with mutations at five *pfdhps *codons, 436, 437, 540, 581, and 613, accounted for 39% of isolates. The CQ resistance-associated mutations *pfcrt *K76T and *pfmdr1 *N86Y rose from 82% to 97% (p = 0.001) and 44% to 76% (p < 0.001), respectively, from baseline to follow-up.

**Conclusions:**

During the period in which SP was the first-line anti-malarial in Kenya, highly SP-resistant parasites emerged, including isolates harboring *pfdhps *mutations not previously observed there. SP continues to be widely used in Kenya; however, given the highly resistant genotypes observed in this study, its use as a first-line anti-malarial should be discouraged, particularly for populations without acquired immunity to malaria. The increase in the *pfcrt *K76T prevalence, despite efforts to reduce CQ use, suggests that either these efforts are not adequate to alleviate CQ pressure in Kisumu, or that drug pressure is derived from another source, such as the second-line anti-malarial amodiaquine.

## Background

In Kenya, a sub-Saharan African nation of 34.7 million people, approximately 70% of the population is at risk for malaria infection, and malaria accounts for approximately 20% of admissions to health facilities and 30-50% of outpatient clinic visits [1]. Effective treatment of malaria in Kenya has been hampered by high levels of drug resistance, both to chloroquine (CQ), which was the first-line anti-malarial for uncomplicated malaria in Kenya until 1998 [2], and to its replacement, sulphadoxine-pyrimethamine (SP). Widespread treatment failures were observed within five years of implementing SP as the first-line anti-malarial, and in 2004 the recommended treatment for uncomplicated malaria was changed to the artemisinin combination therapy (ACT), artemether-lumefantrine (AL) [3]. More than two years elapsed before the training of health care workers and distribution of AL were completed in 2006 [3]. Although no longer the official first-line anti-malarial, SP remains available and is frequently used in the private sector [4]. It also plays an important role in the prevention of malaria in pregnancy when used for intermittent presumptive treatment of malaria in pregnant women (IPTp) [5] and has been included in several trials of intermittent presumptive treatment in infants (IPTi) [6-11].

The efficacy of SP depends on the inhibition of two parasite enzymes necessary for the synthesis and recycling of folate: dihydrofolate reductase (DHFR), the target of pyrimethamine, and dihydropteroate synthase (DHPS), the target of sulphadoxine. The key event in the development of pyrimethamine resistance is a mutation at codon 108 that changes serine to asparagine, resulting in partial pyrimethamine resistance. Further mutation at codons 51 (N51I) and/or 59 (C59R) increases the severity of pyrimethamine resistance [12,13]. An additional mutation, I164L, is associated with high-level SP resistance and has been found in the presence of triple mutant *pfdhfr *in South American and Asian isolates [14,15]. This mutation appears to be rare in Africa [16], but has been detected at low prevalence in isolates from Kisumu and Western Kenya [17-19]. In the background of double or triple mutant *pfdhfr*, mutations that enhance resistance to antifolates arise in the *pfdhps *gene. Five mutations in *pfdhps *(S436A/F, A437G, K540E, A581G, and A613S/T) have been implicated in sulphadoxine resistance [20,21]. *In vivo*, the *pfdhps *double mutant A437G/K540E is associated with clinical SP failure in Africa [13]. Mutations at codons 581 and 613 have begun to emerge in Africa in the background of the A437G mutation [22,23]. These mutations are widespread in South America and South East Asia, and are associated with high-level SP resistance [15,24]. Together, the *pfdhps *double mutant A437G/K540E and the *pfdhfr *triple mutant S108N/N51I/C59R form the *pfdhfr*/*pfdhps *quintuple mutant, which is associated with SP treatment failure [13].

Chloroquine resistance is associated with mutations in two transporters, the chloroquine resistance transporter (PfCRT) and the transmembrane glycoprotein Pgh1, a homolog of the mammalian multidrug resistance transporter encoded by the gene *pfmdr1*. The *pfcrt *mutation K76T is sufficient for CQ resistance [25]. The *pfmdr1 *mutation N86Y is linked with CQ resistance and mefloquine sensitivity [26,27]. In contrast, quinine and mefloquine resistance are associated with parasites that are wild-type at codon 86, but have increased *pfmdr1 *copy number [26]. Among other common *pfmdr1 *polymorphisms, the single mutation N1042D and the allele combination S1034C/N1042D/D1286Y are associated with increased *in vitro *resistance to quinine and increased susceptibility to mefloquine and artemisinin [28]. *In vivo*, treatment with AL selects for parasites that are wild-type at codons 86 and 1246 and mutant at codon 184 [29,30]. In Africa, where CQ has exerted the most drug pressure on these alleles, the *pfcrt *K76T haplotype and single copy *pfmdr1 *with the N86Y haplotype predominate. While both markers are associated with CQ resistance, *pfcrt *K76T is more strongly associated with *in vivo *CQ resistance than either *pfmdr1 *N86Y or both markers together [31]. Research in Malawi has shown that fitness costs imparted by the K76T mutation result in the return of CQ sensitivity in the absence of CQ pressure. In 1993, CQ was replaced as the first-line anti-malarial in Malawi. By 2001, the K76T mutation had completely disappeared, and in 2006, CQ was demonstrated to again be effective *in vivo *[32,33].

In 1999, shortly after SP became the first-line anti-malarial in Kenya, the U.S. Army initiated a study of the prevalence of molecular markers of CQ and SP resistance in Kisumu, Kenya, a malaria endemic area near Lake Victoria. To evaluate changes in molecular markers of SP and CQ resistance in the presence of continued SP selection pressure, and presumably decreased CQ selection pressure, a follow-up study was conducted from 2003-2005. During this time period, the national first-line anti-malarial policy changed from SP to AL due to widespread SP treatment failures, although AL was not fully implemented until after completion of the follow-up study [3]. Here, changes in the prevalence of drug resistance-associated *pfdhfr*, *pfdhps*, *pfcrt*, and *pfmdr1 *haplotypes in the malaria-endemic area of Kisumu, Kenya are compared between the baseline and follow-up studies.

## Methods

### Study site and *Plasmodium falciparum *patient isolates

The goal of this study was to compare the prevalence of molecular markers of drug resistance between the period directly following adoption of SP as the first-line anti-malarial in 1998 and approximately five years later. A baseline study of resistance markers was conducted from 1999-2000 and was reported previously [34]. The findings of a second study conducted from August 2003 through July 2005 are described here and compared to the baseline study results. During both study periods, *P. falciparum *isolates were collected from persons in Kisumu, Kenya, a city located on the shore of Lake Victoria in a region where malaria is holoendemic. Patients of all ages who presented at the outpatient clinics of New Nyanza Provincial Hospital, Kisumu, Kenya, tested positive for malaria by thick smear, and who had not taken anti-malarial drugs within 24 hours were eligible. Informed consent was obtained from all patients, or their parents or guardians. Venous blood for parasite genotyping and *in vitro *drug testing was collected from study subjects prior to anti-malarial treatment. The studies and protocols were approved by the Institutional Review Boards at the Walter Reed Army Institute of Research (WRAIR) and the Kenya Medical Research Institute (KEMRI).

### Genotyping

Blood spots from venous blood collected prior to treatment were dried on filter paper and DNA was isolated by Chelex extraction. To control for variation between the studies, genotype analyses for the baseline and follow-up studies were conducted under identical laboratory conditions by the same personnel, and laboratory strains were included as controls. Parasite genotypes at *pfdhfr *codons 51, 59, 108 (detection of S108N only), and 164, *pfdhps *codons 436, 437, 540, 581, and 613, and *pfcrt *codon 76 were determined by mutation-specific nested PCR. The full-length *pfdhfr *and *pfdhps *genes were amplified in a primary PCR reaction according to the methods of Duraisingh [35] and Wang [36], respectively. Allele specific PCR analyses were performed according to published methods [13]. The genotype of *pfcrt *codon 76 was determined by nested PCR according to the methods described by Djimde and colleagues [31]. The genotypes of *pfmdr1 *codons 86, 184, 1034, 1042, and 1246 were analysed by PCR-RFLP [31]. Genotypes were initially classified as wild-type, mutant, or mixed wild-type and mutant. Isolates were classified as mixed genotype when strong bands of similar intensity were detected for both the wild-type and mutant genotypes. To determine changes in the mutation prevalence at each locus, isolates were grouped as wild-type (pure wild-type only) or mutant (pure and mixed mutant). For each codon, isolates missing data for that codon were excluded from the mutation prevalence analysis.

### *pfdhfr/pfdhps *haplotype analysis

The prevalence of the *pfdhfr *triple mutant allele N51I/C59R/S108N, the *pfdhps *double mutant allele A437G/K540E, and the *pfdhfr*/*pfdhps *quintuple mutant were determined using a modification of the method described by Kublin and colleagues [13]. This method is particularly useful for mixed genotype isolates, such as those observed here. In these isolates, it is difficult to ascertain haplotype because the observed haplotype is a combination of the haplotypes of all the parasite lines in the infection; the method of Kublin and co-workers accounts for this when classifying haplotypes [13].

*Pfdhfr *and *pfdhps *haplotypes were first classified as wild-type, single mutant, double mutant (mixed or pure), or, in the case of *pfdhfr*, triple mutant (mixed or pure) (Additional file 1). These haplotypes were then used to determine the *pfdhfr*/*pfdhps *haplotype (Additional file 2). Although in the published method mutations at codons 51 or 59 were always accompanied by mutant codon 108, several of the Kisumu isolates had mutations at codons 51 and/or 59 but not 108. Thus, in these samples, mutation at codon 108 could not be considered a prerequisite for further *pfdhfr *mutations, and the method was modified to classify isolates with mixed mutations at two *pfdhfr *loci as single mutant (Additional file 1). The published method was further modified by creating a double mutant mixed *pfdhps *category to account for the high number of mixed haplotypes in the samples (Additional file 1).

Due to the high prevalence of *pfdhps *mutations at codons 436, 581, and 613 in the follow-up study, expanded *pfdhps *haplotypes that included these codons were constructed. Using the *pfdhps *A437G/K540E haplotypes (Additional file 1), it was determined whether isolates that were wild-type, single mutant, or double mutant at codons 437 and 540 also carried mutations (mixed and pure mutant combined) at codons 436, 581, and 613. Isolates for which data were missing for any of *pfdhfr *codons 51, 59, or 108 or *pfdhps *codons 436, 437, 540, 581, and 613 were excluded from all haplotype analyses.

### *pfcrt *K76T allele frequency estimation

The frequency of the K76T allele in the baseline and follow-up studies was estimated using the formulas described by Hill and Babiker, assuming a conditional Poisson distribution for the number of clones [37].

### *In vitro *drug susceptibility assays

Red blood cells from venous blood samples taken from smear positive patients were resuspended in folate-free RPMI (Gibco) supplemented with 10% human serum and buffered with HEPES and NaHCO_3_. The culture suspension was added to 96-well plates containing serial dilutions of the anti-malarial drugs sulphadoxine, dapsone, pyrimethamine, chlorcycloguanil, proguanil, chloroquine, mefloquine, quinine, and amodiaquine. The reference strains D2 and W6 were included as controls. Inhibition of parasite growth was assessed by ^3^H-hypoxanthine uptake as previously described [38]. The *in vitro *IC_50 _cutoff values used to classify parasites as drug sensitive or resistant were based on published values determined by the correlation of *in vitro *IC_50 _values with *in vivo *treatment failure (for CQ) and by the statistical definition of resistance as an IC_50 _value greater than two standard deviations above the mean IC_50 _of sensitive parasites (all other drugs). IC_50 _values considered discriminative for resistance were 100 nM (32 ng/ml) for CQ [39]; 800 nM (259.5 ng/ml) for quinine, with 500 nM (162.2 ng/ml) considered a marker of intermediate susceptibility [40]; 30 nM (11.4 ng/ml) for mefloquine; 80 nM (28.5 ng/ml) for amodiaquine [41]; 2 μM (497.4 ng/ml) for pyrimethamine with intermediate levels of susceptibility denoted by IC_50 _values between 500 nM (124.5 ng/ml) and 2 μM [42]; 32 μM (10 μg/ml) for sulphadoxine [43]; and 12 μM for dapsone (3 μg/ml) [44].

### Statistical analysis

Changes in prevalence by study period were analysed by Fischer's exact test if one or more cells contained five or fewer samples, and by the χ^2 ^test for all other analyses. For all analyses, statistical significance was determined at the p < 0.05 level. Statistical analyses were performed with Stata Statistical Software: Release 9.0 (StataCorp, College Station, TX).

## Results

**Figure 1:**
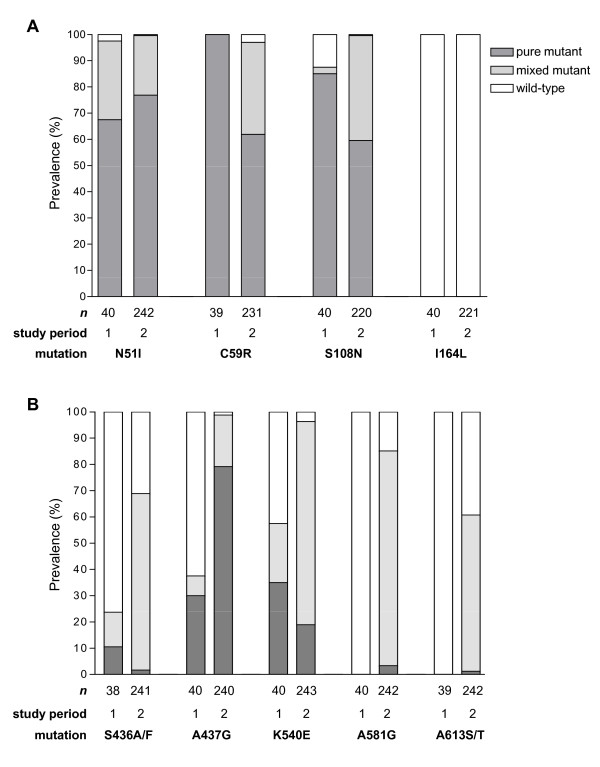
**Prevalence of patient isolates with mutations in (A) *pfdhfr *and (B) *pfdhps *from the baseline study (study period 1) and follow-up study (study period 2)**.

Mutations at four codons in *pfdhfr *and at five codons in *pfdhps *were successfully analysed for 38 to 40 out of 40 total samples from the baseline study (1999-2000) and 220 to 243 out of 250 total samples from the follow-up study (2003-2005). The prevalence of mutant haplotypes (mixed and pure mutant) at each locus was compared between the study periods. In the baseline study [34], the prevalence of patient isolates containing the *pfdhfr *mutations N51I and C59R was high, totaling 97.5% and 100%, respectively, while the prevalence of the mutation S108N was slightly lower at 87.5%. In the follow-up study, the mutation prevalence at codons 51 and 59 was 99.6% and 97.0%, respectively, and did not change significantly from the baseline study (p = 0.14 and p = 0.28, respectively) (Figure 1A). The prevalence of the S108N mutation increased significantly to 99.5% (p < 0.001). Thus, after approximately five years of SP use in Kisumu, over 95% of symptomatic patients had parasites with mutations at *pfdhfr *codons 51, 59, and 108 in either mixed or pure form. The highly drug resistant mutation I164L was not detected in either study period.

The mutations A437G and K540E are the *pfdhps *mutations most strongly associated with SP treatment failure in Africa [13]. The *pfdhps *mutations S436A, A581G, and A613S/T combined with A437G increase the IC_50 _of sulfa drugs in *in vitro *culture relative to A437G alone [20,21], although their role in *in vivo *SP failure is not well understood. The prevalence of A437G and K540E increased from 37.5% to 98.8% (p < 0.001) and from 57.5% to 96.3% (p < 0.001), respectively, between the baseline and follow-up study periods (Figure 1B). The prevalence of S436A/F increased from 23.7% to 68.6% between the study periods (p < 0.001), following the trend of increasing mutation prevalence observed for codons 437 and 540 (Figure 1B). The mutations A581G and A613S/T were not detected in the initial study period; however, these mutations were present in 85.1% (p < 0.001) and 60.7% (p < 0.001) of isolates from the second study period, respectively.

**Figure 2:**
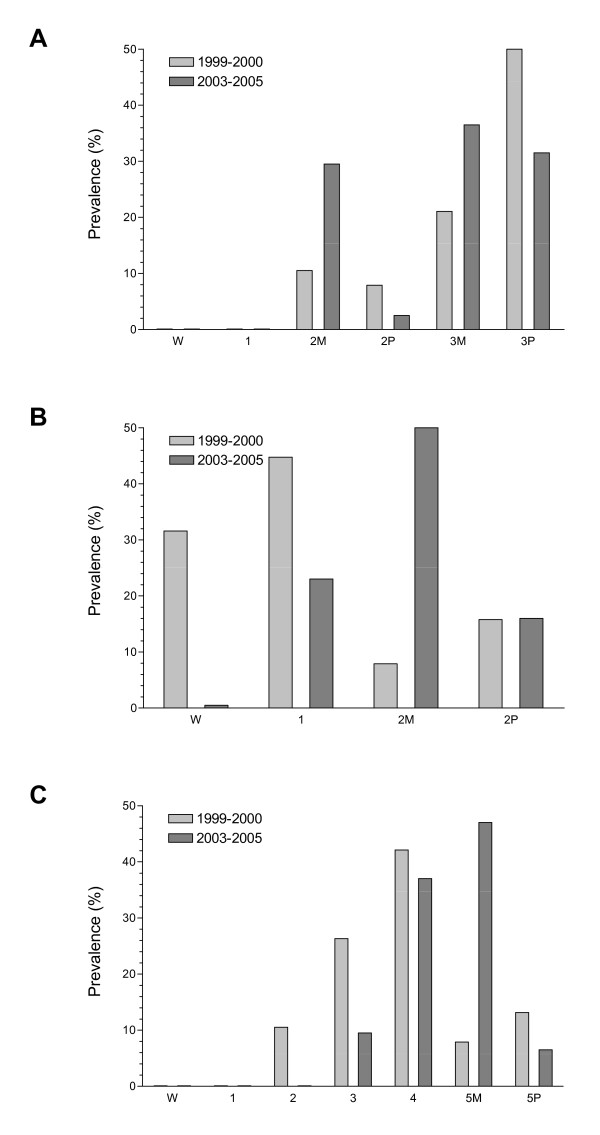
**Prevalence of (A) *pfdhfr*, (B) *pfdhps*, and (C) *pfdhfr*/*pfdhps *haplotypes with respect to *pfdhfr *codons 51, 59, and 108 and *pfdhps *codons 437 and 540 in patient isolates from the baseline and follow-up studies**. Genotypes are wild-type (W), single (1), double (2), triple (3), quadruple (4), and quintuple (5) mutant, with mixed genotypes denoted by M and pure genotypes by P.

Changes in prevalence were also analysed for the three *pfdhfr *and *pfdhps *haplotypes that have been associated with clinical failure of SP: triple mutant *pfdhfr *N51I/C59R/S108N, double mutant *pfdhps *A437G/K540E, and the *pfdhfr*/*pfdhps *quintuple mutant formed from these combined haplotypes. Haplotype analyses were performed on 38 of the 40 isolates from the baseline study and 200 of the 250 isolates from the follow-up study that had complete genotype data for *pfdhfr *codons 51, 59, and 108 and *pfdhps *codons 436, 437, 540, 581, and 613. The prevalence of the *pfdhfr *triple mutant (mixed and pure mutant combined) decreased from 81.5% in the baseline study to 68.0% in the follow-up study, with a coincident rise in the prevalence of *pfdhfr *double mutants from 18.4% to 32.0% (p < 0.001) (Figure 2A). The percentage of isolates containing the *pfdhps *double mutant (mixed and pure combined) increased from 23.7% in the baseline study period to 76.5% in the follow-up period (p < 0.001) (Figure 2B). The prevalence of parasites wild-type at *pfdhps *codons 437 and 540 decreased between the two study periods from 31.6% to 0.5%. The *pfdhfr/pfdhps *quintuple mutant, in either mixed or pure form, is the most clinically relevant molecular marker for SP resistance. The prevalence of mixed and pure quintuple mutant parasites increased from 21.1% to 53.5% in the three to five year interval between studies (p < 0.001) (Figure 2C).

**Figure 3:**
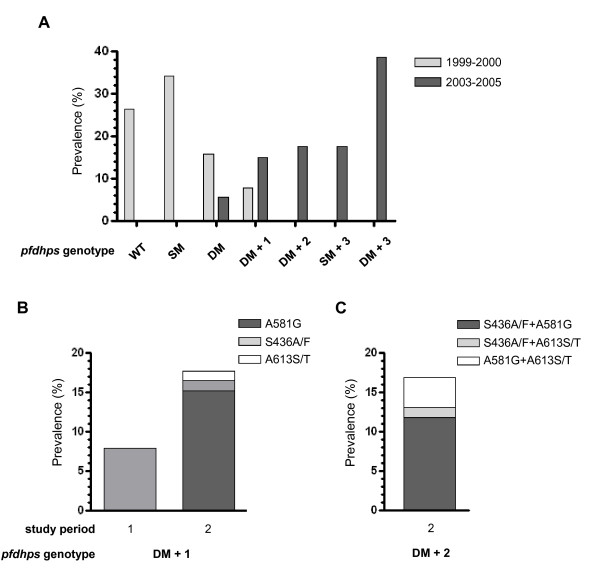
**Prevalence of *pfdhps *haplotypes with respect to codons 436, 437, 540, 581, and 613**. (A) Prevalence of *pfdhps *haplotypes that are wild-type at all five codons (WT), single or double mutant with respect to A437G and K540E (SM or DM), single mutant with three additional mutations at codons 436, 581, and 613 (SM + 3), or double mutant with one, two, or three additional mutations (DM + 1, 2, or 3). (B) Distribution of mutations at codons 436, 581 and 613 in the baseline (study period 1) and follow-up (study period 2) studies among isolates that are A437G/K540E double mutant with one additional mutation. (C) Distribution of mutations at codons 436, 581 and 613 in the follow-up study among isolates that are A437G/K540E double mutant with two additional mutations.

Based on the high prevalence of *pfdhps *mutations at codons other than 437 and 540 in the follow-up study, *pfdhps *genotypes were constructed based on whether isolates that were wild-type, single, or double mutant at codons 437 and 540 had additional mutations at codons 436, 581, and 613. In the baseline study, isolates that were wild-type at codons 436, 581, and 613 and single or double mutant at codons 437 and 540 accounted for 34.2% and 15.8% of isolates, respectively. Only 7.9% of isolates that were A437G/K540E double mutant also had an additional mutation (Figure 3A). In the follow-up study, isolates that were A437G/K540E double mutant with additional mutations in *pfdhps *predominated, such that 14.5% of isolates had one additional mutation, 17.5% had two additional mutations, and 38.5% had three additional mutations (Figure 3A). Only 5.5% contained the A437G/K540E double mutant without additional mutations in *pfdhps*. An additional 17.5% of isolates were single mutant with respect to codons 437 and 540 and carried mutations at codons 436, 581, and 613. In the baseline study, A437G/K540E double mutant isolates were found with one additional mutation, S436A/F; however, in the follow-up study, the majority of isolates with one additional mutation carried A581G (Figure 3B). Among double mutant isolates with two additional mutations, S436A/F and A581G most commonly occurred together (Figure 3C).

In addition to evaluating changes in parasite genotypes associated with drug resistance, *in vitro *IC_50 _values for the common antifolates pyrimethamine, proguanil, chlorcycloguanil, and dapsone were determined for between 27 and 43 isolates from the follow-up study (Table 1). The geometric mean IC_50 _of pyrimethamine was 171.0 ng/ml and the geometric mean IC_50 _of sulphadoxine was 9.3 μg/ml.

**Table 1:** *In vitro *geometric mean IC_50 _data for antifolates in the follow-up study

Drug	*N*	Mean IC_50_ (ng/ml)	Range (ng/ml)	Percent resistant
pyrimethamine	43	171	21-2028	16.3 (53.5)^a^
chlorcycloguanil	41	2	0.5-11	
proguanil	41	929	129-3376	
sulphadoxine	41	9310	890-48,153	51.2
dapsone	27	648	16-129	14.8

^a ^percent resistant using the intermediate level of susceptibility of 500 nM (124.5 ng/ml) as the cutoff

**Figure 4:**
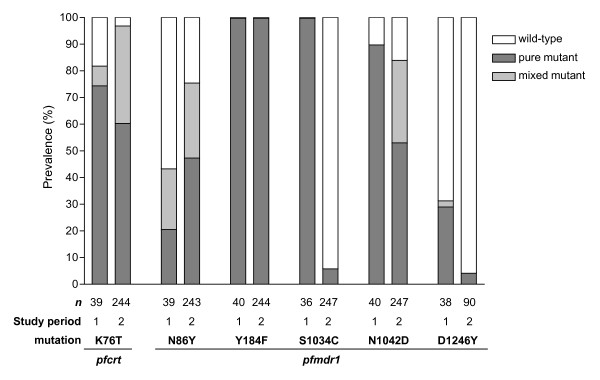
**Prevalence of patient isolates with mutations in *pfcrt *and *pfmdr1 *from the baseline study (study period 1) and follow-up study (study period 2)**.

To evaluate whether changes in quinoline resistance occurred after SP replaced chloroquine as the first-line anti-malarial, the prevalence of mutations in the genes *pfcrt *and *pfmdr1 *were compared between the baseline and follow-up studies. Mutations at *pfcrt *codon 76 and at *pfmdr1 *codons 86, 184, 1034, 1042, and 1246 were successfully analysed in 36 to 40 out of 40 samples from the 1999-2000 study period and 243 to 247 out of 250 samples from the follow-up period (with the exception of *pfmdr1 *codon 1246, for which 90 samples were genotyped). The prevalence of the *pfcrt *mutation K76T in patient isolates increased between the study groups from 82.1% to 97.1% (p = 0.001) (Figure 4). In the *pfmdr1 *gene, significant changes in mutation prevalence were observed at codons 86, 1034, and 1246. The prevalence of the N86Y mutation increased from 43.6% to 75.7% (p < 0.001), while at codons 1034 and 1246 mutation prevalence decreased from 100% to 6.1% (p < 0.001) and 31.5% to 4.4% (p < 0.001), respectively. The mutation prevalence at codon 184 remained at 100% for both the baseline and follow-up studies, and the prevalence of mutant codon 1042 was similar between the baseline and follow-up studies (90.0% and 84.2%, respectively; p = 0.475) (Figure 4).

The prevalence of wild-type *pfcrt *76 has been increasing in coastal Kenya [45] and in Tanzania [46]; therefore, it was important to evaluate whether Kisumu isolates deviated from this trend due to an increase in the frequency of the K76T allele or as a consequence of the higher prevalence of polyclonal infections in the follow-up study compared to the baseline study. The frequency of the K76T allele in the baseline and follow-up studies was estimated using the method of Hill and Babiker [37]. The frequency of K76T was estimated to be 77.8% (63.7%-88.5%) in the baseline study and 85.0% (81.8%-87.9%) in the follow-up study.

IC_50 _values for 54 to 62 samples from the follow-up study were obtained for CQ, mefloquine, quinine, and amodiaquine (AQ) (Table 2). Geometric mean IC_50 _values for CQ, mefloquine, quinine, and AQ were 15.3, 12.0, 47.7, and 7.4 ng/ml, respectively. The prevalence of *in vitro *resistance to CQ, mefloquine, and quinine was also determined. The CQ resistance level is of particular interest because the cutoff of 100 nM for resistant parasites was associated with *in vivo *treatment failure in Cameroon [39], in contrast to other resistance cut-offs that were determined statistically. *In vitro *resistance to CQ was observed in 15% of the isolates; quinine *in vitro *resistance was 1.6%, with 4.9% showing intermediate levels of susceptibility; and 51.6% of isolates were resistant to mefloquine.

**Table 2:** *In vitro *geometric mean IC_50 _data for quinolines in the follow-up study

Drug	*N*	Mean IC_50_ (ng/ml)	Range (ng/ml)	Percent resistant
chloroquine	60	15.3	3-110	15
mefloquine	62	12.0	1-48	51.6
quinine	61	47.7	14-313	1.6 (4.9)^a^
amodiaquine	54	7.4	3-24	0

^a ^percent resistant using the intermediate level of susceptibility of 500 nM (162.2 ng/ml) as the cutoff

## Discussion

The first-line anti-malarial treatment regimen for uncomplicated malaria in Kenya changed from CQ to SP in 1998 and from SP to AL in 2004. In both instances, the change was necessitated by widespread treatment failure. The prevalence of SP and chloroquine resistance-associated genotypes and *in vitro *resistance was compared between the period just after SP implementation (1999-2000) and a period five to seven years later (2003-2005) in patients with febrile illness presenting to outpatient clinics in Kisumu, Kenya. Malaria in Kisumu and in other areas surrounding Lake Victoria is endemic. For the 12 months ending in June 2004, the entomological inoculation rate in Kisumu district was 31.1 infective bites per person per year [47], and in the time period 2003-2006, the median *P. falciparum *parasite rate for children 2 to 10 years of age (PfPR_2-10_) was 71% in Kisumu [48]. Despite changes in government anti-malarial policy, as late as 2010 SP was used to treat malaria in 37% of households surveyed in Kisumu, compared to 32% that used ACT [4].

The prevalences of *pfdhfr *codons N51I, C59R, and S108N were near saturation in the baseline study [34] and remained relatively stable between the baseline and follow-up studies. In contrast, the prevalence of mutations at all five *pfdhps *codons analysed increased dramatically over the same period. It thus appears that a significant proportion of isolates carried *pfdhfr *mutations prior to 1998, and use of SP as the first-line anti-malarial in Kenya resulted in the development of highly mutant *pfdhps*. Pre-existing mutations in *pfdhfr *may have derived from a combination of two sources: the use of SP as a second-line anti-malarial in Kenya prior to 1998 and the ongoing treatment of persons with HIV/AIDS with co-trimoxazole, a bacterial DHFR/DHPS inhibitor used to treat respiratory tract infections and prevent opportunistic infections. Co-trimoxazole shows cross-resistance with pyrimethamine and sulphadoxine in *in vitro P. falciparum *culture [49,50] and may play a role in the development of mutations in *pfdhfr *and *pfdhps*.

One goal of this study was to determine whether the prevalence of the clinically relevant *pfdhfr/pfdhps *quintuple mutant, which is associated with SP treatment failure in Africa [13,51], changed during the course of SP use as the first-line anti-malarial in Kenya. Between the baseline and follow-up studies, the prevalence of the quintuple mutant doubled to 53.5% and may be even higher. Due to absence of MOI data in these studies, *pfdhfr/pfdhps *haplotypes were deduced according to the method described by Kublin and colleagues [13]. Because this method conservatively classifies isolates that are pure mutant at one *pfdhfr *allele and mixed at two other *pfdhfr *alleles as double mutant, the prevalence of the *pfdhfr *triple mutant may be systematically underestimated in populations where mixed infections are common. In the follow-up study, many more isolates had mixed *pfdhfr *genotypes than at baseline, and the prevalence of the *pfdhfr *triple mutant decreased between the baseline and follow-up studies. This observation contrasts with other findings showing that the population of SP-resistant parasites expanded over this period, and may be explained by more severe systematic underestimation in the follow-up study compared to the baseline. Thus, the prevalence of the *pfdhfr *triple mutant and the *pfdhfr/pfdhps *quintuple mutant in the follow-up study may actually be higher than estimated here.

Although the prevalence of the *pfdhfr *triple mutant appeared to decrease over time, increases in the prevalence of the *pfdhps *A437G/K540E double mutant led to a rise in the *pfdhfr/pfdhps *quintuple mutant prevalence. The very high prevalence of mutant codons 437 and 540 is consistent with other reports that these mutations are common in western Kenya [19,52]. Notably, the data presented here show that SP use in Kenya was not just associated with the expansion of A437G and K540E, but also the progressive accumulation of mutations in *pfdhps *at codons 436, 581, and 613. This process appears to be rapid, as demonstrated by the emergence and spread of the A581G and A613S/T mutations in the short time between the baseline and follow-up studies.

Mutations at *pfdhps *codons 436, 581, and 613 are associated with increased levels of *in vitro *resistance when they occur with the A437G mutation [20,43]. Among isolates in the follow-up study, over half had the mutations S436A/F, A581G, and A613S/T in addition to the A437G/K540E single or double mutant; A437G was present in either mixed or pure form in all single mutant isolates. Thus, at follow-up the majority of isolates had parasite genotypes associated with extremely high *in vitro *sulphadoxine resistance. Although the effect of these mutations *in vivo *is not well-studied, under SP selection, parasites containing the quintuple mutant and A581G have been found to have a selective advantage *in vivo *over quintuple mutant parasites [53]. This selective advantage is reflected in the swift development and expansion of *pfdhps *mutations between the baseline and follow-up studies.

This is the first study to report the presence of the A581G mutation in Kenya. Although relatively rare in sub-Saharan Africa, this mutation has been documented in the neighboring countries of Uganda and Tanzania [19]. In the background of mutant *pfdhfr *in Tanzania, a similar increase in the prevalence of A437G and K540E accompanied by the emergence and rapid spread of A581G was observed over the six year period following SP implementation [46]. Notably, in this and other East African studies that reported the presence of A581G, all or most samples were wild-type at codon 613 [46,53,54]. This contrasts sharply with the findings reported here, where mutant codon 613 was present in over half of patient isolates in the follow-up study. It thus appears that the mutation A581G is becoming increasingly common in East Africa, while Kisumu may be a regional hotspot for the mutation A613S/T.

Although progressive mutations in *pfdhps *were observed, the *pfdhfr *mutation I164L, which is associated with high levels of chlorcycloguanil and pyrimethamine resistance in South America and South East Asia, was absent from isolates in both study periods. This mutation was detected in isolates from Kisumu in 2002 [17], prior to the start of the follow-up study, and in western Kenya in 2004 [18]. The absence of the I164L mutation in isolates from the follow-up study, conducted from 2003-2005, indicates that parasites harboring this mutation in 2002 did not become widespread in Kisumu. Taken together, these findings are consistent with evidence that the I164L mutation is rare in sub-Saharan Africa, outside local hotspots in south-west Uganda and Rwanda [16,19,55].

The change from CQ to SP as the first-line anti-malarial in 1998 was expected to decrease CQ use in Kenya, possibly leading to restoration of CQ sensitive parasites. In Malawi, discontinuing the use of CQ led to the reversion of codon 76 to wild-type over a seven year period, and CQ sensitivity *in vivo *was restored after 12 years [32,33]. Trends of increasing prevalence of wild-type codon 76 have recently been reported in other areas of East Africa. In Tanzania, the prevalence of wild-type *pfcrt *codon 76 increased over a six year period after anti-malarial policy replaced CQ with SP [46]. Surveillance in coastal Kenya from 1993-2006 showed that the wild-type codon 76 prevalence also increased, although at a slower rate than in Malawi [45]. Therefore, it was surprising to observe a statistically significant increase in the prevalence of isolates containing *pfcrt *K76T from 82% to 97%, as well as a significant increase in the prevalence of *pfmdr1 *N86Y, also associated with chloroquine resistance, from 44% to 76%. To analyse whether the apparent increase in the prevalence of mutant codon 76 arose from the higher prevalence of polyclonal infections observed in the follow-up study, the method of Hill and Babiker was used to estimate the frequency of the K76T allele. Even accounting for polyclonal infections, there was an apparent although not statistically significant increase in the frequency of K76T. Thus, the K76T mutation does not appear to be abating in Kisumu, as it is in coastal Kenya and in other areas in East Africa.

The persistence of the *pfcrt *K76T mutation in isolates from a high transmission setting such as Kisumu indicates that selection for the mutant codon is ongoing. The *pfcrt *K76T mutation is associated with amodiaquine as well as chloroquine treatment failure [56]. Although AQ was officially the second-line therapy used in Kenya during the study period, it was available at 95% of drug retail outlets, whereas SP was available at only 29% of outlets [57]. By comparison, CQ was stocked by 15% of drug retail outlets [57]. It thus appears that widespread use of AQ in the private sector may be high enough to exert selective pressure on the parasite population.

Specific *pfmdr1 *genotypes have been linked to altered susceptibility to artemisinin, a component of the current first-line anti-malarial therapy AL. *In vitro*, the allele combination S1034C/N1042D/D1246Y is associated with increased artemisinin susceptibility [28], and *in vivo*, treatment with AL selects for the N86, Y184F, and D1246 alleles [29,30]. In this study, the prevalence of alleles associated with artemisinin sensitivity (S1034C and D1246Y) decreased significantly; however, this decline is not likely due to selective pressure induced by AL as completion of the follow-up study predated widespread use of AL. Whether the prevalence of *pfmdr1 *mutations continues to change under increasing AL pressure will be of interest.

## Conclusions

During the period in which SP was used as the first-line anti-malarial in Kenya, the prevalence of clinically relevant molecular markers of SP and CQ resistance increased in Kisumu, a city located on the shore of Lake Victoria in a malaria endemic region. Mutations at *pfdhps *codons 581 and 613, which have not previously been observed in Kenya, became highly prevalent during this period. In light of these highly SP-resistant genotypes and reports that SP remains widely used in Kenya [4], it is important to discourage use of SP to treat symptomatic malaria, particularly for populations that are not semi-immune. Furthermore, whether the *pfdhfr/pfdhps *quintuple mutant combined with the *pfdhps *mutations S436A/F, A581G, and A613S/T adversely impacts the efficacy of IPTp and IPTi should be evaluated. The continued rise in the prevalence of mutations associated with CQ resistance indicates that continued drug pressure, either from CQ or amodiaquine use, is preventing restoration of CQ-susceptible parasites in Kisumu. That these findings contrast with a study covering the same period in coastal Kenya [45] highlights the importance of surveillance and the heterogeneity in drug resistance that may occur within a country.

## Competing interests

The authors declare that they have no competing interests.

## Authors' contributions

MDS participated in the design of the study, analysed the data, and wrote the manuscript. FE and HA performed the molecular analyses. RLC, SAB, and NCW conceived of and coordinated the studies. RLC and NCW wrote the protocol. STP assisted in writing the manuscript. WJM helped design the study, analyse the data, and write the manuscript. All authors read and approved the final manuscript.

## Supplementary Material

Additional file 1 **Classification of *pfdhfr *and *pfdhps *genotypes**. White boxes indicate wild-type genotype, gray boxes indicate mixed mutant genotype, and black boxes indicate pure mutant genotype. Adapted from [13]. Click here for file

Additional file 2 **Classification of *pfdhfr*/*pfdhps *haplotypes using the genotype classifications in Additional file ****1**. Table showing *pfdhfr*/*pfdhps *haplotype classifications. Click here for file
