# Supplementary material for: Increased prevalence of the pfdhfr/phdhps quintuple mutant and rapid emergence of pfdhps resistance mutations at codons 581 and 613 in Kisumu, Kenya
Source: Malar J. 2010 Nov 24;9:338. doi: 10.1186/1475-2875-9-338 (PMC3001743; doi:10.1186/1475-2875-9-338)

Additional file 1

DHFR Codon
51 59 108

Wild-type

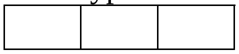

Single

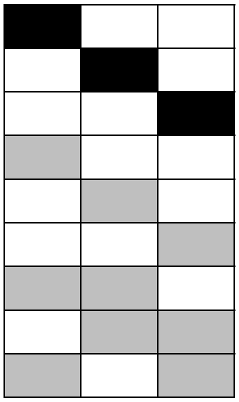

Double mixed

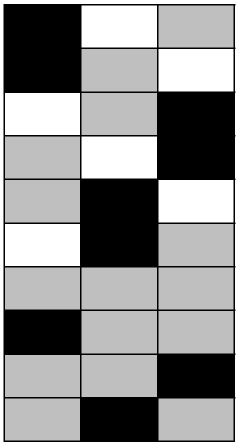

Double pure

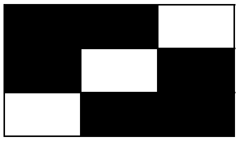

Triple mixed

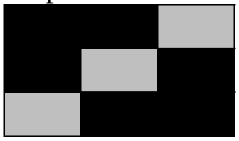

Triple pure

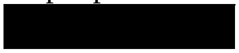

DHPS Codon
437 540

Wild-type

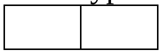

Single

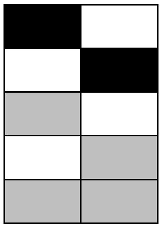

Double mixed

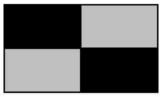

Double pure

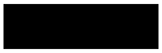

Supplement: Additional file 1 — Classification of pfdhfr and pfdhps genotypes. White boxes indicate wild-type genotype, gray boxes indicate mixed mutant genotype, and black boxes indicate pure mutant genotype. Adapted from [13]. [file 1475-2875-9-338-S1.PDF]
