# Supplementary material for: Increased prevalence of the pfdhfr/phdhps quintuple mutant and rapid emergence of pfdhps resistance mutations at codons 581 and 613 in Kisumu, Kenya
Source: Malar J. 2010 Nov 24;9:338. doi: 10.1186/1475-2875-9-338 (PMC3001743; doi:10.1186/1475-2875-9-338)
Supplement: Additional file 2 — Classification of pfdhfr/pfdhps haplotypes using the genotype classifications in Additional file 1. Table showing pfdhfr/pfdhps haplotype classifications. [file 1475-2875-9-338-S2.DOC]

**Additional file 2.** Classification of *pfdhfr*/*pfdhps* haplotypes using the genotype classifications in Additional file 1.

| **DHFR genotype** | **DHPS genotype** | **DHFR/DHPS genotype** |
| --- | --- | --- |
| wild-type | wild-type | wild-type |
| wild-type | single | single |
| single | wild-type | single |
| wild-type | double (mixed or pure) | double |
| double (mixed or pure) | wild-type | double |
| single | double (mixed or pure) | triple |
| double (mixed or pure) | single | triple |
| triple (mixed or pure) | wild-type | triple |
| triple (mixed or pure) | single | quadruple |
| double (mixed or pure) | double (mixed or pure) | quadruple |
| triple (mixed or pure) | double (mixed or pure) | quintuple mixed |
| triple pure | double pure | quintuple pure |
